# Interbody fusion in degenerative lumbar spinal stenosis with additional posterolateral fusion using *Escherichia coli*-derived bone morphogenetic protein-2: A Pilot study

**DOI:** 10.1097/MD.0000000000020477

**Published:** 2020-06-12

**Authors:** Sung Hoon Choi, Ja Wook Koo, DaeHyun Choe, Jeong Min Hur, Dong-Hong Kim, Chang-Nam Kang

**Affiliations:** Department of Orthopedic Surgery, Hanyang University College of Medicine, Seoul, Republic of Korea.

**Keywords:** *E coli*-derived Bone morphogenetic protein-2, lumbar spine, posterolateral fusion, spinal fusion, spinal stenosis

## Abstract

This case series investigated the efficacy and optimal dose of *Escherichia coli*-derived bone morphogenetic protein-2 (E.BMP-2) as a bone graft substitute for additional posterolateral spinal fusion, accompanying interbody fusion procedures, for treating lumbar degenerative spinal stenosis. This study focused on the optimal dose for each segment and the efficacy of E.BMP-2 as a substitute for autogenous iliac bone graft.

Ten patients were enrolled from January 2015 to December 2015, and underwent an additional posterolateral fusion procedure, with 2.5 mg of E.BMP-2 followed by decompression, transpedicular fixation, and interbody fusion. The mean follow-up period was 13.9 months, and regular radiological examinations were performed in every case. Clinical outcomes were measured with a visual analog scale for back pain (VAS-BP), and leg pain (VAS-LP) and the Korean Oswestry Disability Index (K-ODI). All parameters were assessed preoperatively and postoperatively at 12 months.

All 18 segments treated with E.BMP-2 completely fused in 6 months as observed on both simple radiography and computed tomography. The mean fusion period was 4.5 months on simple radiography. At 12 months follow-up, VAS-BP, VAS-LP, and K-ODI scores (1.9 ± 1.5, 1.9 ± 1.9, 11.0 ± 6.6, respectively) had improved significantly compared to preoperative scores (5.5 ± 1.9, 6.5 ± 1.9, and 49.9 ± 11.5, respectively, *P* < .05). There were no postoperative wound infections, neurological symptoms, or complications associated with the use of E.BMP-2 during the follow-up period.

E.BMP-2 could be used to enhance the outcomes in posterolateral spinal fusion following interbody fusion surgery. In the present study, 2.5 mg of the E.BMP-2 per segment was sufficient to obtain bony union in posterolateral fusion surgery. Further large-scale trials with long-term follow-up are necessary to evaluate the various complications related to the use of E.BMP-2.

## 1 Introduction

Autogenous iliac bone remains the most common grafting material used in spinal fusion procedures. Although the fusion rate following use of autogenous iliac bone is high, 25% to 30% of patients experience donor site complications, and nonunion has been reported in 5% to 35% cases.^[1,2]^ Recently, efforts have been made to enhance fusion rates by mechanical reinforcement to achieve firm fixation during spinal fusion. However, such efforts have failed to eliminate the issues of nonunion. In addition, there is a need for alternative grafting materials in cases where the use of autogenous iliac bone is impossible or in the presence of other problems. Therefore, in order to overcome the limitations of autogenous iliac bone grafting, substitute materials have received much attention.^[3–6]^

Bone morphogenetic protein-2 (BMP-2) is a growth factor that belongs to the transforming growth factor (TGF) superfamily. BMP-2 binds to TGF-type receptors that transduce signals, prompting gene expression mainly through the Smad pathway and has been employed clinically.^[7–10]^ Chinese hamster ovary cell-derived recombinant human BMP-2 (C.BMP-2) has been widely researched as a bone graft substitute; however, the low yield and high cost for obtaining sufficient amounts of the graft remains problematic. To overcome these shortcomings, *Escherichia coli*-derived recombinant human BMP-2 (E.BMP-2) was introduced, and few studies have reported the clinical outcomes in the field of odontology and animal experimentations.^[11–16]^ However, there are no guidelines regarding the adequate dosage of E.BMP-2 in additional posterolateral fusion following posterior interbody fusion of the spine. In addition, most studies on bone grafting materials that contain bioactive derivatives of E.BMP-2 have relied on animal tests rather than human subjects.^[11,15,16]^

The purpose of this study was to determine the effectiveness of E.BMP-2 as a bone graft substitute and the appropriate dose for each segment when performing additional posterolateral spinal fusion, accompanying interbody fusion procedures, in the treatment of lumbar degenerative spinal stenosis.

## 2 Materials and methods

This retrospective cohort study investigated ten patients (2 males and 8 females) who underwent 1 or 2-segment decompression, transpedicular fixation, and interbody fusion for degenerative lumbar disease between January and December 2015. The patients also underwent additional posterolateral fusion using 2.5 mg of E.BMP-2 (NOVOSIS, CGBIO, Korea). The mean follow-up period was 13.9 months, and the age of patients at the time of surgery varied from 60 to 81 years, with a mean of 68.2 years.

Inclusion criteria were intractable low back pain, radiculopathy, or neurogenic claudication despite more than 3 months of conservative treatment including medication, physiotherapy, and selective nerve root block. All E.BMP-2 used in this study were provided free of charge by the transfer contract of material after obtaining informed consent from the patients. Pure spinal stenosis was observed in 2 cases (patients #2 and #6); while the other presentations included patients with spinal stenosis in addition to degenerative scoliosis (patients #1 and #3), isthmic spondylolisthesis (patient #4), degenerative spondylolisthesis (patient #5), herniated intervertebral disc (patients #8 and #9), segmental instability (patient #10), and herniated intervertebral disc with segmental instability (patient #7).

All patients underwent open transforaminal lumbar interbody fusion with additional posterolateral fusion using 2.5 mg of E.BMP-2 with hydroxyapatite granules (HA) as a carrier in each segment on the left or right side. The fixed segments were L3-5 in 3 cases, L4-5 in two cases, and L4-S1 in 5 cases, and none of the patients underwent re-operation (Table 1). There were a total of 18 fixed segments, and E.BMP-2 was used on an average in 1.8 segments.

**Table 1:**
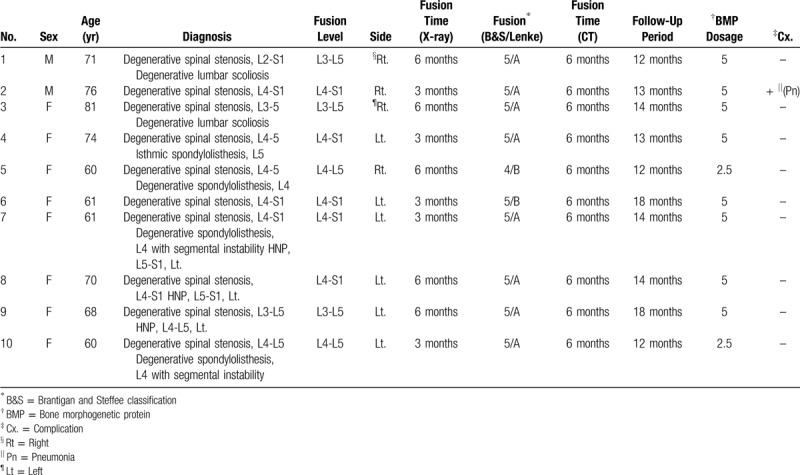
Clinical data of the patients.

Plain radiographs were taken immediately after surgery, 1 month, 3 months, 6 months, and 12 months after the surgery, whereas computed tomography (CT) images were taken at 6 months and 12 months after the surgery. All cases were followed up for at least 12 months, and fusion was assessed based on plain radiographs and CT imaging 1 year after the surgery. For evaluating the quality and quantity of the fused mass, grade 4 or higher according to the Brantigan and Steffee classification of interbody fusion^[17,18]^ were considered “union” on CT, and Grades A and B according to the Lenke classification^[19]^ of posterolateral fusion were considered “union” on plain radiographs.

Scores of the visual analogue scale for back pain (VAS-BP), visual analogue scale for radiating pain of the lower extremity (VAS-LP), and the Korean Oswestry Disability Index (K-ODI) were compared before and 12 months after the surgery to assess the effect of treatment, and complications during the follow-up period were evaluated. Preoperative and postoperative radiological and clinical evaluations were compared using the Wilcoxon signed-rank test in SPSS version 20.0 (IBM Co., Armonk, NY). *P* values <.05 were considered statistically significant.

The complications examined were manifestations of heterotopic ossification, osteolysis, seroma/hematoma, postoperative wound infections, neurological symptoms, and retrograde ejaculation, which are known adverse effects of BMP.^[20]^ To minimize the bias and determine non-inferiority of the E.BMP-2 compared to a previously reported study, the sample size was calculated to obtain a power of 80% with an alpha of 0.05, under the circumstances of 35% estimation of the pseudarthrosis rate of posterolateral fusion. According to this consideration, seven patients were required, and finally, ten were enrolled in anticipation of a 20% follow-up loss. The first author who was not involved in the index surgery measured all the radiographical and clinical scales.

## 3 Results

**Figure 1:**
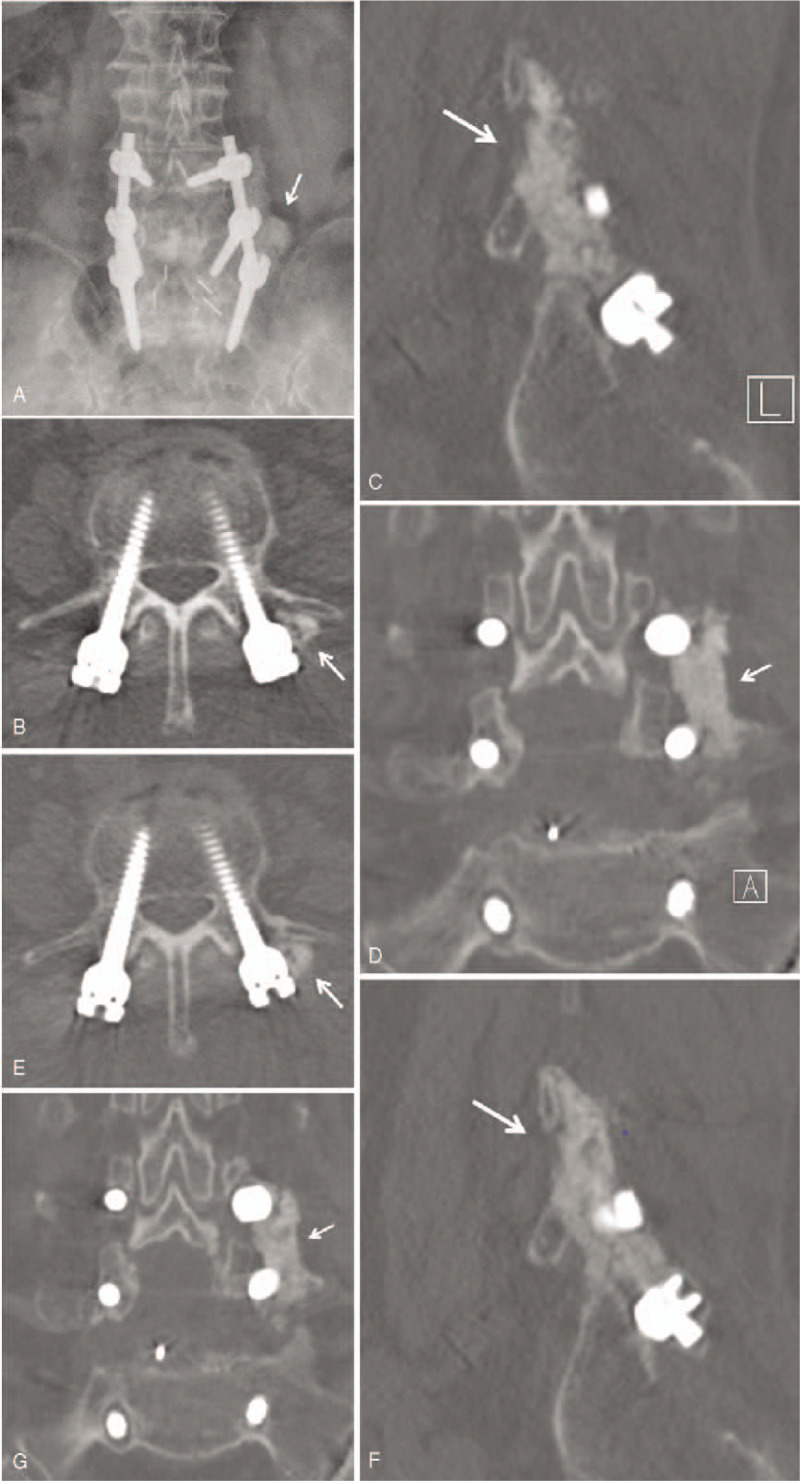
(A) A 76-year-old woman underwent decompression, transpedicular fixation, interbody fusion, and posterolateral fusion for degenerative spinal stenosis in L4-5, and isthmic spondylolisthesis (L5 on S1). Antero-posterior plain radiographs taken 6 months after surgery showed grade A fusion according to the Lenke classification on the left side (arrow). (B–D) Axial (B), sagittal (C), and coronal (D) CT images obtained 6 months after surgery showed grade A solid fusion. (E, F, G) Axial (E), sagittal (F), and coronal (G) CT images obtained 12 months after surgery showed grade A solid fusion.

The demographic data of patients included in this study are presented in Table 1. E.BMP-2 was applied to either side of the segment in posterolateral fusion, to the right side in four cases and the left side in 6 cases. E.BMP-2 was applied to 18 segments in total and complete fusion was achieved in all. On plain radiography, 5 patients showed solid fusion of grade A and B according to the Lenke classification at 3 months after surgery, and all patients showed solid fusion 6 months after the surgery, with a mean of 4.5 months. Eight cases were classified as Grade A and 2 as grade B 6 months after the surgery (Fig. 1A–G). And all cases were classified as Grade A according to the Lenke classification based on the CT images taken 12 months after the surgery.

At 12 months follow-up, VAS-BP, VAS-LP, and K-ODI scores (5.5 ± 1.9, 6.5 ± 1.9, and 49.9 ± 11.5, respectively,) had improved significantly compared with preoperative scores (1.9 ± 1.5, 1.9 ± 1.9, and 11.0 ± 6.6, respectively, *P* < .05, Table 2).

**Table 2:**
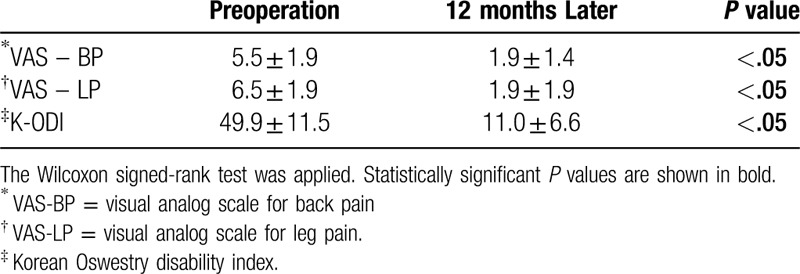
Clinical outcomes.

There were no postoperative wound infections, neurological symptoms, or complications related to instrumentation. One patient underwent medical treatment due to postoperative pneumonia; was administered intravenous antibiotics (piperacillin/tazobactam 4.5 g, q 8 hourly) for 2 weeks, and was discharged following recovery. There were no complications reflecting the adverse effects of E.BMP-2 or diagnoses of malignant tumors during the follow-up period.

## 4 Discussion

In 1911, Hibbs^[21]^ and Albee^[22]^ used spinal fusion to treat Pott disease and published their first clinical report. Since then, fusion-related surgical techniques and instrumentation have advanced greatly. Autogenous iliac bone grafting is considered the gold standard in spinal fusion. However, the technique has limitations in cases of revision surgery and where the use of autogenous iliac bone (history of radiation therapy, fracture around the iliac bone, among others) is challenging. Hence, several studies have investigated various types of bone grafting materials as substitutes capable of osteoinduction and osteoconduction to increase fusion rates by regenerating bone and achieving suitable mechanical strength.^[3–6,23]^ Boden et al^[5,6,10]^ reported that osteoconductive materials must be used along with BMP as an osteoinductive material. In addition, many studies have reported use of growth factors following the advancement of molecular biology, including BMP variants, insulin-like growth factor-1 and -2, platelet-derived growth factor, and demonstrated that only BMP variants are capable of heterotopic bone formation.

Vaidya et al^[24]^ compared the results of C.BMP-2 and autogenous iliac bone grafts in spinal fusion and reported fusion rates of 100% and 92.3%, respectively at 180 days and 274 days, respectively. Bodalia et al^[25]^ reported that C.BMP-2, which shortens the fusion period, showed better results in improving pain in a 2-year follow-up study and resulted in a fusion rate of 88.1% in revision surgery. Taghavi et al^[26]^ studied patients who underwent revision surgery for pseudarthrosis and reported a 100% fusion rate in cases where C.BMP-2 was used. The adverse effects of C.BMP-2 with excellent outcomes in spinal fusion have been studied. Known adverse effects of the protein include heterotopic ossification, osteolysis, seroma/hematoma, infections, neurological symptoms (myelopathy, radiculopathy), and retrograde ejaculation.^[20]^ Mulconrey et al^[27]^ reported a subfacial hematoma that required surgical drainage as a postoperative complication in one of 26 patients treated with C.BMP-2. Beachler et al^[28]^ studied the use of C.BMP-2 and the risk of malignant tumors using the Surveillance, Epidemiology, and End Results-Medicare system from 2004 to 2011 and found no correlation between the use of C.BMP-2, incidence of malignant tumors, and the resultant mortality. In our study, no patient was diagnosed with malignant tumors during the follow-up period.

C.BMP-2 has been widely used in spinal surgery since it yields outcomes similar to autogenous bone, enables more rapid fusion, and demonstrates high success rates even in revision surgery or multi-segment fusions. However, the clinical utilization of C.BMP-2 has been limited in South Korea because of its low yield and high cost for obtaining sufficient amounts of the graft. Recently, a study compared the safety and efficacy of E.BMP-2 and autogenous iliac bone graft in posterolateral fusion surgery.^[29]^ This study evaluated clinical outcomes in patients who underwent decompression, transpedicular fixation, and interbody fusion for degenerative lumbar disease, as well as additional posterolateral fusion with 2.5 mg of E.BMP-2 in one segment. As shown in Table 1, all segments were fused in the postoperative 6 months CT without any complications related to the use of E.BMP-2. Interestingly, 50% of the patients showed Grade A and B solid fusion on the 3 months postoperative plain radiographs and 100% solid fusion on the 6 months postoperative CT scan. Cho et al also reported 100% solid fusion on the 12 weeks postoperative CT with E.BMP-2 and HA granules in posterolateral fusion surgery.^[29]^ This suggests rapid fusion capacity of E.BMP-2 in the heterotopic ossification of the spine; therefore, further analysis with a larger sample size and longer follow-up are needed to further assess the fusion rate in similar patients.

The present study is significant as it is the first in South Korea that reported 1-year follow-up results of the use of E.BMP-2 in the additional posterolateral fusion surgery subsequent to interbody fusion. The fact that 2.5 mg of E.BMP-2 was effective in one segment when posterolateral fusion was performed is important in future studies. The limitations of this study include the small sample size without a control group. In this study, we focused on the non-inferiority of E.BMP-2 compared to previously reported data on pseudarthrosis, and ten patients were sufficient to demonstrate the outcomes. Additionally, the possible impact of the E.BMP-2 carrier on the fusion rate may have been overlooked. Lastly, the short follow-up period could not thoroughly estimate the various complications associated with the use of E.BMP-2.

This study confirmed that E.BMP-2 could be used to enhance the outcomes of posterolateral spinal fusion surgery. In the present study, 2.5 mg of E.BMP-2 per segment was sufficient to obtain bony union in the additional posterolateral fusion procedure. Further large-scale trials with long-term follow-up are necessary to evaluate the various complications related to the use of E.BMP-2.

## Author contributions

**Conceptualization:** Chang-Nam Kang.

**Data curation:** Ja Wook Koo.

**Formal analysis:** Ja Wook Koo, DaeHyun Choe, Jeong Min Hur.

**Investigation:** Ja Wook Koo, Dong-Hong Kim.

**Methodology:** Sung Hoon Choi.

**Project administration:** Sung Hoon Choi.

**Software:** DaeHyun Choe, Jeong Min Hur.

**Supervision:** Chang-Nam Kang.

**Validation:** Chang-Nam Kang, Sung Hoon Choi.

**Visualization:** Sung Hoon Choi.

**Writing – original draft:** Sung Hoon Choi, Chang-Nam Kang.

**Writing – review & editing:** Sung Hoon Choi, Chang-Nam Kang.
